# Diagnostic Accuracy for Periprosthetic Joint Infection Does Not Improve by a Combined Use of Glucose and Leukocyte Esterase Strip Reading as Diagnostic Parameters

**DOI:** 10.3390/jcm11112979

**Published:** 2022-05-25

**Authors:** Marco Haertlé, Louisa Kolbeck, Christian Macke, Tilman Graulich, Ricarda Stauß, Mohamed Omar

**Affiliations:** Department of Trauma Surgery, Hannover Medical School, Carl-Neuberg-Str. 1, 30625 Hannover, Germany; louisa.kolbeck@pius-hospital.de (L.K.); macke.christian@mh-hannover.de (C.M.); graulich.tilman@mh-hannover.de (T.G.); stauss.ricarda@mh-hannover.de (R.S.)

**Keywords:** orthopedics, diagnostics, arthroplasty, periprosthetic joint infection, Combur test, urine strip test

## Abstract

The diagnosis of periprosthetic infections (PJI) can be challenging in clinical practice because the clinical presentations of aseptic loosening and low-grade infections are similar. Semiquantitative evaluation of leukocyte esterase (LE) in synovial fluid using a urine strip test has already established itself as a diagnostic method over the past decade. The analysis of LE in synovial fluid leads to a high number of false-positive test results. In the present study, the value of a combined semiquantitative determination of glucose and LE in synovial fluid to improve the diagnosis of PJI was investigated. Over a 4-year period, 145 synovial samples were collected from patients who developed joint effusion after arthroplasty. LE and glucose test strips were considered as an index test for the diagnosis of PJI. A ++ or +++ LE and a negative glucose test strip reading were considered as positive test results. Modified diagnostic criteria for PJI as recommended by the Musculoskeletal Infection Society (MSIS) served as the reference test, except that intraoperative findings were excluded. Forty-six out of 145 samples were classified as septic complication according to the reference test. In regard to PJI, our data showed that combined use of LE and glucose strip test reading displayed a 98.0% specificity (95% confidence interval (CI): 95.2% to 100%), a 50% sensitivity (95% CI: 35.6% to 64.4%), a 92% positive predictive value (95% CI: 81.4% to 100.0%), and an 80.3% negative predictive value (95% CI: 73.2% to 87.4%). In contrast, the exclusive analysis of LE on the urine strip to diagnose PJI demonstrated a 90.9% specificity (95% CI: 85.2% to 96.6%), a 67.4% sensitivity (95% CI: 53.8% to 80.9%), a 77.5% positive predictive value (95% CI: 64.6% to 90.4%), and an 85.7% negative predictive value (5% CI: 79.0% to 92.4%). A combination of LE and glucose test pad reading is considered superior as a potential “rule-in” method for the diagnosis of PJI compared with LE test pad analysis alone. However, combined LE and glucose synovial fluid testing also demonstrated lower test sensitivity and thus diagnostic accuracy compared with LE analysis alone. Therefore, combined glucose and LE test pad analysis does not represent a sufficient diagnostic standard to exclude PJI with certainty.

## 1. Introduction

Colorimetric reagent strip tests are a diagnostic tool for detecting infections in various body fluids by detecting LE associated with the presence of white blood cells [1,2]. Previous studies have investigated the role of reagent strip tests, thereby establishing the rapid synovial fluid test as a diagnostic method for septic arthritis (SA) and PJI. Based on these results, the reagent strip test also became part of common practice to aid in the diagnosis of PJI [3,4]. Apart from septic complications, nonseptic inflammatory joint diseases are thought to be associated with increased leukocyte concentration and, therefore, lead to false-positive test results when synovial fluid is examined for LE alone. Omar et al., reported that analysis of synovial glucose levels using the reagent strip test or a glucometer provides an additional diagnostic tool to distinguish between different inflammatory states of native joints [5,6]. A normal synovial sample contains approximately 3.3–5.3 mmol/L glucose, which corresponds to the glucose level in human plasma [7]. Microbial metabolism reduces the glucose concentration in the synovial fluid and can therefore provide additional diagnostic information [8].

With regard to PJI, there is no evidence that the combined determination of glucose and LE on the reagent strip increases diagnostic specificity and sensitivity. Therefore, we conducted this study to investigate whether the combined measurement of glucose and LE on the colorimetric reagent strip improves the diagnostic accuracy in PJI compared with measuring glucose or LE alone. In this study, we wanted to investigate whether the simultaneous measurement of LE and glucose significantly reduces the number of false-positive test results due to nonseptic joint conditions.

## 2. Materials and Methods

We conducted this diagnostic study over a period of 4 years (2014–2017). After the study protocol was reviewed and approved by the local ethics committee, patient recruitment took place in our emergency department and outpatient clinic. The planned sample size was not determined before the start of the study. As soon as patients who had undergone arthroplasty presented to our department with joint effusion, enrollment in this study was assessed. The inclusion criteria were the presence of an artificial joint with joint effusion and the age of the patient being more than 18 years. Patients with traumatic joint effusion were excluded from this study before joint aspiration was performed. In addition, patients who had an inadequate volume of synovial fluid drained or the joint aspirate analyzed more than 6 h after joint puncture were considered ineligible. A total of 26 patients were excluded based on the above criteria.

All study participants signed an informed consent form before arthrocentesis. Synovial fluid was collected by alternating investigators under sterile conditions using an 18-gauge needle. The collected samples were analyzed with LE and glucose strip assays. Synovial fluid cell counts and microbial incubation using cultures and staining procedures were performed. In addition, the percentage of polymorphonuclear leukocytes (PMN) in the synovial fluid was examined. Furthermore, blood samples were obtained from the participants and the levels of serum glucose (s-glucose), serum C-reactive protein (s-CRP), and peripheral blood leukocytes (PBLs) were evaluated. The integrity of the data was retrospectively reviewed. 

The analysis of glucose and LE by colorimetric reagent strip test was treated as an index test. The presence of LE in liquids results in a purple color change due to a hydrolytic reaction that occurs when the liquid sample contacts the chemicals on the reagent strip [4]. Glucose triggers a semiquantitative color change on the test pad due to a reaction with the enzyme glucose oxidase, which leads to the formation of gluconic acid, thereby reducing atmospheric oxygen to hydrogen peroxide [9]. For semiquantification of LE and glucose in joint effusions, one drop of synovial fluid was applied to each test pad of a urine strip (Combur; Roche, Grenzach-Wyhlen, GER). Test results were read after 60 s by the investigator in charge. Inconclusive test results of the reagent strip due to blood contamination of the aspirated synovial fluid were prevented by using a minicentrifuge protocol (Omnilab, Bremen, GER). After 60 s of centrifugation, samples were separated into a solid red cell pellet and a synovial fluid supernatant [10]. Then the supernatant was collected and applied to the test area of the reagent strip. As reference for the colorimetric change, we used the provided scale on the dipstick packaging. The results for the LE test are displayed in the range “−” (0 cells/mm^3^) to “+++” (>500 cells/mm^3^). In the same manner, the semiquantitative glucose analysis ranges from “−” (0 mmol/L) to “++++” (55 mmol/L). According to the data previously published by Parvizi et al., the LE values “-” and “+” were considered as negativity cut-off [11]. With respect to glucose concentration, no change of color at the test pad represented a diminished amount of glucose in the synovial fluid [5]. 

In daily practice, diagnostic criteria defined by the MSIS are commonly used to diagnose PJI. According to this diagnostic tool, (1) the presence of a sinus tract communicating with the prosthesis, (2) the detection of identical pathogens isolated from two or more aspirate cultures, or (3) a cumulative score of supportive criteria greater than or equal to six entailing serum-CRP, serum D-dimer, ESR, elevated synovial fluid WBC count, LE reading (++/+++), synovial alpha-defensin, synovial PMN percentage, synovial CRP and intraoperative findings including positive histology and the presence of pus or positive microbial growth may be considered definitive evidence of PJI. Based on the literature, the MSIS criteria affirm a sensitivity of 97.7% and a specificity of 99.5% [3].

Collected samples not matching the MSIS diagnostic requirements were classified aseptic. Not all patients underwent surgical treatment, so intraoperative findings, which are also part of the MSIS classification, were not available for all patients. Therefore, these intraoperative criteria were not applied to the classification of our samples.

Three diagnostic constellations were evaluated as index tests: (1) a positive (++ or +++) LE test, (2) a negative glucose (−) test, and (3) a combination of a positive LE test (++ and +++) and a negative glucose (−) test. Sensitivity, specificity, positive predictive value, negative predictive value, positive likelihood ratio, negative likelihood ratio, and area under the curve (AUC) were calculated for each test arrangement. For statistical analysis of data between study groups, we used analysis of variance or the Kruskal–Wallis test. Values are expressed as mean and 95% confidence interval (CI). *p*-Values of <0.05 were considered significant. Statistical analysis was performed using GraphPad Prism^®^ software (version 9.0.0 (86); GraphPad Software, San Diego, CA, USA) and Microsoft^®^ Excel (version 16.43, Microsoft, Redmond, WA, USA). 

## 3. Results

One hundred forty-five patients were enrolled in the study. The study population consisted of 74 female and 71 male patients. A total 46 study participants were diagnosed with PJI. The septic cohort had a mean age of 67 (±15.7) years. Male patients accounted for 69.6% of all participants diagnosed with PJI. The aseptic group had a mean age of 70 (±10.9) years. Females accounted for 60.6% of aseptic complications in this study. Arthrocentesis in the PJI group was performed on 26 knee joints (56.5%), 19 hip joints (41.3%), and 1 elbow (2.2%) joint. Aseptic samples of synovial fluid were obtained from 72 knee joints (73.7%), 25 hip joints (24.7%), 1 shoulder joint (1.0%), and 1 elbow (1.0%) joint (Table 1). 

Analysis of the collected blood and synovial fluid samples was performed in the manner described above and revealed significantly higher levels of serum CRP, synovial WBCs, and synovial PCM percentage in the septic cohort (Table 2). 

Forty-two of the 46 synovial fluids classified as septic showed microbial growth in culture. The most commonly detected pathogens were Staphylococcus strains, e.g., *Staph. aureus* and *Staph. epidermidis* (Table 3). 

Thirty-one (67.4%) samples classified as septic had a ++ or +++ semiquantitative leukocyte esterase test reading, while nine (9.0%) aseptic probes displayed a ++ or +++ leukocyte esterase test reading. In general, a significantly positive LE test reading was associated with an increased number of synovial leukocytes and PMN regardless of the study group (Table 4).

Twenty-three (53.5%) septic synovial fluid samples had a combined negative glucose and positive LE test reading. Within the aseptic cohort, the combination of a negative glucose and positive LE drip test reading was seen twice (2.0%). Further cross tabulation was performed for different combinations regarding LE and glucose reagent strip test results (Table 5). 

In our study, the LE test reading alone as a diagnostic parameter for PJI had a sensitivity of 67.4% (95% CI: 53.5% to 80.9%) and a specificity of 90.1% (95% CI: 85.2% to 96.6%), resulting in nine false-positive test results. The combined use of a positive LE and a negative glucose strip test result as an index test resulted in an increase in specificity to 97.9% (95% CI: 95.2% to 100.0%), leading to an increase in positive predictive value to 92.0% (95% CI: 81.4% to 100.0%). The negative predictive value decreased to 80.3% (95% CI: 73.2% to 87.4%). 

**Figure 1:**
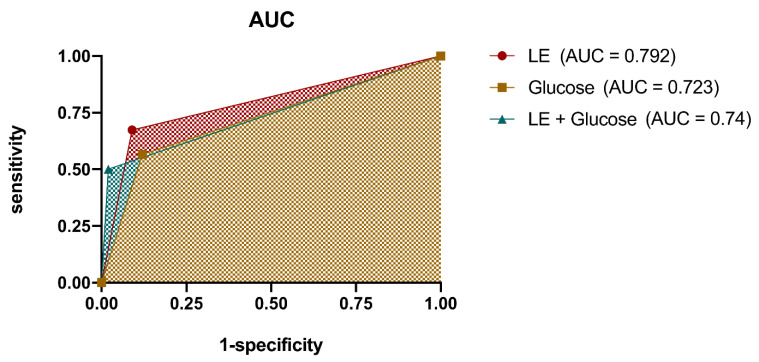
Area under the curve calculation of the receiver operating characteristic curves visualize the diagnostic accuracy of the differ- ent diagnostic pathways. LE (Leukocyte esterase).

As a result of the increased specificity, the sensitivity of the combined glucose and LE urine test reading decreased to 50.0% (95% CI: 35.6% to 64.4%), resulting in 23 false-negative test results. Taken together, semiquantification by enzymatic colorimetric LE testing alone showed the leading diagnostic accuracy at 79.2% (95% CI: 71.0% to 87.4%) compared with glucose alone or combined glucose and LE test reading (Table 6, Figure 1).

## 4. Discussion

In 2011, the idea of using LE analysis after arthrocentesis to aid in the diagnosis of PJI was introduced by Parvizi et al. [12]. Since then, several studies have investigated the diagnostic accuracy of the LE reagent strip test in the context of PJI. In our cohort, evaluation of the LE test reading showed a sensitivity of 67.39% and a specificity of 90.90%. The results of our semiquantitative LE analysis were consistent with the increases in leukocyte count and PMN content in synovial fluid and thus appear plausible (Table 4). According to a meta-analysis of 13 studies published by Lin et al., the enzymatic colorimetric LE test has a pooled sensitivity of 79% (95% CI: 75%–82%) and a pooled specificity of 96% (95% CI: 95%–97%) [13]. Almost all study cohorts included in the meta-analysis used MSIS criteria to classify septic specimens. Five of the 13 studies analyzed had a sample size of more than 100 participants. Although the majority of our study samples in our cohort were evaluated according to MSIS criteria, full evaluation according to MSIS could not be performed in patients without surgical care. A large study by Guenther et al., which included 364 patients before arthroplasty revision, calculated a test sensitivity of 100% and a test specificity of 96.5% [14]. The values seem to differ from the data we collected, but both study designs have some clear differences. Guenther et al., considered bacterial growth in synovial cultures as a diagnostic reference standard rather than evaluating the MSIS criteria. In addition, they performed arthrocentesis intraoperatively, in contrast to sample collection in our study. Intraoperative joint aspiration could increase sample quality and avoid false-negative test results. In addition, the authors excluded patients with a history of antibiotic treatment, whereas in our study cohort, inclusion was independent of antibiotic therapy. Although it has been previously reported that prior antibiotic use does not significantly affect test results of the colorimetric reagent strip LE test, antibiotic use may still lead to false-negative results in other MSIS parameters, such as ESR and CRP, thus affecting the results of our study [15]. In 2017, another recent key publication by Parvizi et al., evaluated the test performance of LE urine strip tests and therefore analyzed 659 synovial specimens [16]. Reviewing the largest study cohort in the current literature, the authors reported a test sensitivity of 75% and a test specificity of 90.9% for LE test strip reading. The study results appear to be consistent with our calculations. The authors also used MSIS criteria to classify septic specimens. However, in contrast to our study, all data were collected retrospectively

Overall, the major publications provide similar evidence for the test performance of enzymatic colorimetric LE analysis for the diagnosis of PJI, raising the question of whether combined LE and glucose measurement improves diagnostic performance. 

According to our data, assessment of glucose in synovial aspirate alone resulted in a test sensitivity of 56.62% and a test specificity of 87.87%. With a calculated test accuracy of 72.26%, the isolated analysis of the glucose strip had the weakest diagnostic performance in the context of a PJI diagnosis due to the high number of false-positive and negative test results (Table 6). The value of the synovial glucose level test in the evaluation of septic joint disease has been evaluated previously. A pioneering paper by Vikerfors et al., outlined that decreased glucose levels were more likely in septic joint inflammation than in aseptic disease conditions, such as gout. However, the authors concluded that diagnostic relevance is limited because of false-positive and false-negative test results. Fluctuations in synovial glucose levels in response to food intake or as a result of diabetes mellitus were discussed as possible causes for the lower diagnostic accuracy [17]. Omar et al., published data that glucose measurement with a glucometer should be considered as an additional diagnostic for the evaluation of atraumatic joint effusion with respect to SA in native joints. However, the data showed that synovial glucose measurement by a glucometer for the diagnosis of SA had a relatively low positive predictive value (68.2%), again consistent with our data (Table 6). Therefore, the authors concluded that synovial glucose measurement should be considered only as an additional diagnostic tool to exclude SA [6].

Based on the described results, Drago et al., performed the only available prospective study investigating the diagnostic accuracy of enzymatic colorimetric glucose analysis for the diagnosis of PJI. The results of our study appear to be consistent with the study data of Drago et al., because the study size and inclusion criteria of both studies are comparable. For the study cohort analyzed by Drago et al., a test sensitivity of 77.8%, a test specificity of 81%, and a positive predictive value of 52.5% were calculated [18]. The major disadvantage of synovial glucose analysis appears to be the consistently high number of false-positive and false-negative test results, resulting in decreased diagnostic accuracy.

Many conditions other than PJI are thought to result in decreased glucose levels in the synovial fluid of native and artificial joints. Therefore, some argue that synovial glucose levels must be viewed in relation to blood glucose levels to be diagnostic [19]. In our study cohort, there was no significant difference in serum glucose levels between the septic and aseptic cohorts, so there was no evidence that hyperglycemia had an effect on the amount of glucose in the synovial fluid (Table 2). 

The extent to which the timing in the course of PJI or the type of germ virulence plays a critical role in the measurement of synovial fluid glucose cannot be answered at present and requires further investigation. Synovial glucose metabolism in native joints compared with artificial joints has not been adequately explored yet, so further research is also needed to optimize the diagnostic value and use of synovial glucose determination.

Finally, we evaluated joint colorimetric analysis of glucose and LE test readings in the participant’s joint aspirates with respect to a PJI. Our calculations revealed that the combined assessment of LE and glucose increased the relevance of positive test results as a diagnostic tool. The specificity and negative predictive values of the test increased to 97% and 92%, respectively. The increased number of false-negative test results represented the most negative effect of this study group, resulting in a decrease in test sensitivity and ultimately diagnostic accuracy (Table 6). 

The poor and variable test performance of the glucose strip reagent can be cited as a major reason for this observation. In addition, enzymatic colorimetric LE evaluation showed a relatively high number of false-negative test results (Table 4). The exact cause for this test performance needs further investigation.

Omar et al., were among the first to investigate the joint evaluation of LE and glucose with the reagent strip test as a diagnostic approach for the diagnosis of SA. In contrast to our data, the authors reported that simultaneous measurement of glucose and LE improved diagnostic accuracy and increased the positive predictive value from 34.6% to 94.4%, with no significant impact on test sensitivity and negative predictive value [5]. Apart from the fact that Omar et al., analyzed test performance in native joints, no detailed information on participant characteristics was available to explain the variance in synovial glucose and LE apart from septic reasons. The virulence of the germs detected in both study cohorts appeared to be similar and, therefore, cannot account for the variation in test performance (Table 3). 

The previously mentioned research paper by Drago et al., does not provide statistical data on the joint colorimetric analysis of glucose and LE. To our knowledge, there is no publication analyzing the simultaneous LE and glucose strip test reading as a rapid test method for the diagnosis of PJI. 

The present study has several limitations. The major limitation is the lack of sample size calculation, so the study results must be interpreted cautiously in terms of confirming or rejecting the hypothesis [20]. However, compared with other studies examining the diagnostic performance of reagent strips in the context of PJI, our study had a similar number of participants. 

In addition, not all patients underwent surgical treatment, and therefore, information regarding interoperative findings, which are also part of the MSIS classification, is not available for all participants.

Another important caveat, which has been described previously, is that test results from reagent strips may be affected by contamination of the samples with blood. We eliminated admixed erythrocytes by centrifugation according to the protocol. Centrifugation can potentially cause clumping of leukocytes, leading to falsified test results of the reagent strip [21]. Unfortunately, no data are available on how many samples were centrifuged during the course of our study. 

Moreover, test results were interpreted by multiple investigators, which could affect the reproducibility of test results. Patients were prospectively enrolled in the study, but the complete data collection regarding the number of synovial cells and laboratory values was performed retrospectively, resulting in a higher number of incomplete data sets. As regards our study, the number of septic samples compared with aseptic samples is not representative of the prevalence of the disease. By including a large number of patients with PJI, we were able to make an optimal estimate of the sensitivity and specificity of the test. The downside of this calculation is that the positive and negative predictive values cannot be properly evaluated when prevalence is biased.

## 5. Conclusions

The combined use of LE and glucose strip test reading in the context of confirming the diagnosis of PJI does not show higher diagnostic accuracy than LE strip test reading alone. Nevertheless, diagnostic evaluation of LE and glucose can be considered as an additional rule-in parameter supporting the diagnosis of PJI when a septic joint condition is suspected. In no case does a negative glucose test result exclude the diagnosis of PJI. 

## Figures and Tables

**Table 1:** Demographics and clinical data of the study population.

Characteristics	Infected	Uninfected	*p*-Value
Ø Age (years)			
	67 ± 15.67	70 ± 10.99	
Sex			<0.001
Male	32 (69.57%)	39 (39.39%)	
Female	14 (30.43%)	60 (60.61%)	
Type of affected joint			0.2119
Knee	26 (56.52%)	72 (73.73%)	
Hip	19 (41.30%)	25 (24.7%)	
Shoulder	0 (0.00%)	1 (1.01%)	
Elbow	1 (2.17%)	1 (1.01%)	

**Table 2:** Synovial cell and blood cell analysis comparing septic and aseptic patients.

Characteristics	Septic	Aseptic	*p*-Value
Blood			
Serum CRP (mg/L)	185.99 (12 to 416) *n* = 44	48.16 (0.3 to 312) *n* = 76	<0.00001
PBL (1000/µL)	10.53 (2.2 to 24.3) *n* = 44	8.75 (1.5 to 24.3) *n* = 76	0.05551
S-glucose (mmol/L)	6.68 (3.3 to 16.7) *n* = 38	6.94 (2.6 to 14.8) *n* = 33	0.826
Synovial Fluid			
WBC (1/µL)	53,750.00 (225 to 370,000) *n* = 33	1661.84 (100 to 55,400) *n* = 76	<0.00001
PMN (%)	89.55 (6 to 99) *n* = 29	39.79 (0 to 99) *n* = 72	<0.00001

**Table 3:** Microbacterial data of synovial fluid culture.

Synovial Fluid Culture	No. of Affected Joints
Streptococcus	
*Streptococcus dysgalactiae equisimilis*	1
*Streptococcus agalactiae*	2
*Streptococcus dysgalactiae*	2
*Streptococcus mitis*	1
Staphylococcus	
*Staphylococcus aureus* (*ORSA*)	14 (2)
*Staphylococcus epidermidis*	11
*Staphylococcus lugdunensis*	2
*Staphylococcus haemolyticus*	1
*Propionibacterium acnes*	2
*Sphingomonas paucimobilis*	1
*Escherichia coli*	3
*Enterokokken*	
*Enterococcus faecalis*	2
Haemophilus	
*Haemophilus parainfluenzae*	2
*Candida albicans*	1
Negative	3

**Table 4:** Correlation of synovial fluid characteristics and strip test results.

	LE Neg (*n* = 65)	LE + (*n* = 40)	LE ++ (*n* = 22)	LE +++ (*n* = 18)	*p*-Value
Leukocytes (cell/mm^3^) (no. with complete data)	1320.67 (100 to 33,300) (*n* = 52)	2691.96 (100 to 23,000) (*n* = 28)	41,150.0 (450 to 139,200) (*n* = 16)	84,430.77 (1350 to 370,000) (*n* = 13)	<0.00001
PMN (%) (no. with complete data)	38.65 (0 to 99) (*n* = 50)	51.88 (0 to 96) (*n* = 26)	81.14 (10 to 99) (*n* = 14)	90.25 (62 to 99) (*n* = 12)	<0.00001
Septic sample (no. (% of septic samples))					
Glc neg	1 (2.17)	2 (4.35)	10 (21.74)	13 (28.26)	
Glc 1+	3 (6.52)	5 (10.87)	4 (8.7)	2 (4.35)	
Glc 2+	1 (2.17)	2 (4.35)	1 (2.17)	1 (2.17)	
Glc 3+	0 (0.00)	0 (0.00)	0 (0.00)	0 (0.00)	
Glc 4+	0 (0.00)	1 (2.17)	0 (0.00)	0 (0.00)	
Aseptic sample (no. (% of aseptic samples))					
Glc neg	10 (10.10)	0 (0.00)	1 (1.01)	1 (1.01)	
Glc 1+	17 (17.17)	12 (12.12)	4 (4.04)	0 (0.00)	
Glc 2+	24 (24.24)	14 (14.14)	1 (1.01)	1 (1.01)	
Glc 3+	7 (7.07)	3 (3.03)	1 (1.01)	0 (0.00)	
Glc 4+	2 (2.02)	1 (1.01)	0 (0.00)	0 (0.00)	

LE = leukocyte esterase strip test, Glc = glucose strip test, neg = negative, PMN = polymorphonuclear leukocytes.

**Table 5:** Strip test results in relation to patient diagnosis.

	Diagnosis (No. of Patients)	
Test result	PJI	Aseptic complication
LE ++ or +++		
Yes	31	9
No	15	90
GLC-		
Yes	26	12
No	20	87
LE ++ or +++ and GLC-		
Yes	23	2
No	20	97

LE = leukocyte esterase strip test, GLC = glucose strip test, PJI = periprosthetic joint infection, neg = negative.

**Table 6:** Test accuracy for the diagnosis of PJI using strip test analysis.

	LE ++ or +++	GLC-	LE ++ or +++ and GLC-
Sensitivity (95% CI)	0.6739 (0.5384/0.8094)	0.5652 (0.4219/0.7085)	0.5 (0.3555/0.6444)
Specificity (95% CI)	0.9090 (0.8523/0.9656)	0.8787 (0.8144/0.943)	0.9797 (0.9519/1.0074)
Youden index	0.7649	0.6865	0.5203
Positive predictive value (95% CI)	0.775 (0.6455/0.9044)	0.6842 (0.5120/0.8564)	0.92 (0.8137/1.0263)
Negative predictive value (95% CI)	0.8571 (0.7901/0.924)	0.8131 (0.7392/0.887)	0.8033 (0.7321/0.8744)
Positive likelihood ratio (95% CI)	7.4055 (3.13/11.66)	4.6595 (1.23/8.09)	24.6305 (17.62/31.64)
Negative likelihood ratio (95% CI)	0.3971 (0.3175/0.4767)	0.4948 (0.4134/0.5762)	0.5104 (0.4290/0.5917)
AUC (95% CI)	0.792 (0.7097/0.8743)	0.7226 (0.6318/0.8134)	0.74 (0.6510/0.829)
False positives	9	12	2
False negatives	15	20	23

PJI = Periprosthetic joint infection, LE = leukocyte esterase strip test, GLC = glucose strip test, AUC = Area under the curve, CI = confidence interval.

## Data Availability

All data supporting reported results are stored in the computer center of the Hannover Medical School.

## Abbreviations

CI	confidence interval
CIJD	chronic inflammatory joint disease
CRP	C-reactive protein
ESR	erythrocyte sedimentation rate
LE	leukocyte esterase
MSIS	Musculoskeletal Infection Society
PBL	peripheral blood leucocyte
PMN	polymorphonuclear leukocytes
PJI	periprosthetic joint infection
SA	septic arthritis
WBC	white blood cell

## References

[1] Devillé W.L.J.M., Yzermans J.C., Van Duijn N.P., Bezemer P.D., Van Der Windt D.A.W.M., Bouter L.M. (2004). The urine dipstick test useful to rule out infections. A meta-analysis of the accuracy. BMC Urol..

[2] Azoulay E., Fartoukh M., Galliot R., Baud F., Simonneau G., Le Gall J.R., Schlemmer B., Chevret S. (2000). Rapid diagnosis of infectious pleural effusions by use of reagent strips. Clin Infect. Dis..

[3] Parvizi J., Tan T.L., Goswami K., Higuera C., Della Valle C., Chen A.F., Shohat N. (2018). The 2018 Definition of Periprosthetic Hip and Knee Infection: An Evidence-Based and Validated Criteria. J. Arthroplast..

[4] Sharma K., Ivy M., Block D.R., Abdel M.P., Hanssen A.D., Beauchamp C., Perry K.I., Rosemark C.L., Greenwood-Quaintance K.E., Mandrekar J. (2020). Comparative analysis of 23 synovial fluid biomarkers for hip and knee periprosthetic joint infection detection. J. Orthop. Res..

[5] Kolbeck L., Haertlé M., Graulich T., Ettinger M., Suero E.M., Krettek C., Omar M. (2021). Leukocyte Esterase and Glucose Reagent Test Can Rule in and Rule out Septic Arthritis. In Vivo.

[6] Omar M., Reichling M., Liodakis E., Ettinger M., Guenther D., Decker S., Krettek C., Suero E.M., Mommsen P. (2017). Rapid exclusion of bacterial arthritis using a glucometer. Clin. Rheumatol..

[7] Terčič D., Božič B. (2001). The Basis of the Synovial Fluid Analysis. Clin. Chem. Lab. Med..

[8] Ward P.C.J. (1980). Interpretation of synovial fluid data. Postgrad. Med..

[9] Bekhit M., Wang H.-Y., McHardy S.F., Gorski W. (2020). Infection Screening in Biofluids with Glucose Test Strips. Anal. Chem..

[10] Aggarwal V.K., Tischler E., Ghanem E., Parvizi J. (2013). Leukocyte Esterase from Synovial Fluid Aspirate: A Technical Note. J. Arthroplast..

[11] Tischler E.H., Cavanaugh P.K., Parvizi J. (2014). Leukocyte Esterase Strip Test: Matched for Musculoskeletal Infection Society Criteria. J. Bone Jt. Surg. Am..

[12] Parvizi J., Walinchus L., Adeli B. (2011). Molecular Diagnostics in Periprosthetic Joint Infection. Int. J. Artif. Organs.

[13] Chen Y., Kang X., Tao J., Zhang Y., Ying C., Lin W. (2019). Reliability of synovial fluid alpha-defensin and leukocyte esterase in diagnosing periprosthetic joint infection (PJI): A systematic review and meta-analysis. J. Orthop. Surg. Res..

[14] Guenther D., Kokenge T., Jacobs O., Omar M., Krettek C., Gehrke T., Kendoff D., Haasper C. (2014). Excluding infections in arthroplasty using leucocyte esterase test. Int. Orthop..

[15] Shahi A., Alvand A., Ghanem E., Restrepo C., Parvizi J. (2019). The Leukocyte Esterase Test for Periprosthetic Joint Infection Is Not Affected by Prior Antibiotic Administration. J. Bone Jt. Surg. Am..

[16] Shahi A., Tan T.L., Kheir M., Tan D.D., Parvizi J. (2017). Diagnosing Periprosthetic Joint Infection: And the Winner Is?. J. Arthroplast..

[17] Söderquist B., Jones I., Fredlund H., Vikerfors T. (1998). Bacterial or Crystal-associated Arthritis? Discriminating Ability of Serum Inflammatory Markers. Scand. J. Infect. Dis..

[18] De Vecchi E., Villa F., Bortolin M., Toscano M., Tacchini L., Romano C.L., Drago L. (2016). Leucocyte esterase, glucose and C-reactive protein in the diagnosis of prosthetic joint infections: A prospective study. Clin. Microbiol. Infect..

[19] Brannan S.R., Jerrard D.A. (2006). Synovial fluid analysis. J. Emerg. Med..

[20] Gupta K.K., Attri J.P., Singh A., Kaur H., Kaur G. (2016). Basic concepts for sample size calculation: Critical step for any clinical trials!. Saudi J. Anaesth..

[21] Coiffier G., Pollet S., Albert J.-D., Perdriger A., Guggenbuhl P., Chales G. (2013). Usefulness and limitations of rapid urine dipstick testing for joint-fluid analysis. Prospective single-center study of 98 specimens. Jt. Bone Spine.

